# Breast cancer cells exhibits specific dielectric signature *in vitro* using the open-ended coaxial probe technique from 200 MHz to 13.6 GHz

**DOI:** 10.1038/s41598-019-41124-1

**Published:** 2019-03-18

**Authors:** Mousa Hussein, Falah Awwad, Dwija Jithin, Husain El Hasasna, Khawlah Athamneh, Rabah Iratni

**Affiliations:** 1 Department of Electrical Engineering, College of Engineering, UAE University, United Arab Emirates University, Al Ain, P.O. Box 15551 United Arab Emirates; 2 Department of Biology, College of Science, UAE University, United Arab Emirates University, Al Ain, P.O. Box 15551 United Arab Emirates

## Abstract

Here we investigated the feasibility of using microwave spectroscopy for characterization of normal and breast cancer cell lines cultured *in vitro*. Healthy non-tumorigenic, MCF-10A and breast cancer, MDA-MB-231, Hs578T, T47D and MCF-7 cell lines were electrically characterized using the open-ended coaxial probe technique from 200 MHz to 13.6 GHz. The dielectric constant, dielectric loss and conductivity between breast non-tumorigenic and breast cancer cells lines were analyzed and their differences determined. Our results showed that the four breast cancer cell lines analyzed exhibited higher dielectric properties when compared to healthy cells. Interestingly, we found that breast and colon cancer cells have different dielectric properties as well, thus suggesting that each type of cancer has a unique microwave signature. This study shows that microwave characterization of breast cancer cell lines is reliable with potential in biomedical applications such as designing electromagnetic models for detection of tumorous cells in healthy tissues.

## Introduction

Cancer originates when certain genetic abnormalities arise in a cell or group of cells. Such cells do not respond to normal signaling system of the body and hence divide and multiply uncontrollably forming bulk cancerous tissues. Cancer can be classified depending on the type of cell in which they originate. Breast, lung and colorectal cancers are the most common cancer types with highest incidence rates worldwide.

The increased incidence of cancer is a subject of major concern worldwide. Timely diagnosis of cancer can help in more effective treatment and reduction in mortality rate. Microwave based cancer diagnosis and treatment is one of the most relevant areas of research nowadays. Such research stems from the basic knowledge about the considerable contrast in the dielectric parameters of normal and cancerous tissues^1,2^. The larger dielectric properties of cancerous tissues arise mainly from its increased water content and can contribute to the increased scattering of microwaves. An extensive amount of studies has been carried out to electrically characterize living tissues using microwave spectroscopy techniques^3–17^. Some of the earliest dielectric studies using open ended coaxial probe method were performed on animal tissues. Joines *et al*. measured electrical properties of rat tissues from 30 MHz to 2 GHz^3^, whereas Stuchly *et al*. characterized *ex vivo* cat tissue samples from 0.01 to 1 GHz^4^. The dielectric studies were later extended to human tissues. Joines *et al*. reported dielectric characterization of different types of human tissues *ex vivo* from 50 to 900 MHz^5^. It was estimated that the contrast in permittivity and conductivity between malignant tissues and normal tissues was 6.4:1 and 3.8:1, respectively. Lazebnik *et al*. performed the most extensive study to characterize breast tissues from 0.5 to 2 GHz^11,12^. His experiments unveiled the variation of breast tissue dielectric properties with respect to breast heterogeneity and adipose content. Martellosio *et al*. successfully evaluated breast tissue dielectric properties over a broad frequency range up to 50 GHz^15–17^.

In our studies, we experimented a different approach in which dielectric measurements are performed on cultured cell lines as reported in^18^. Cancer cell lines are frequently used as *in vitro* models of cancer and serve as powerful tools in learning cancer biology and testing cancer treatments. The main advantage of using cell lines is that when grown in a standard medium, they provide an unlimited supply of self-replicating homogeneous cell population. To a good extent, cell lines preserve the genomic properties of the original tissues from which they have been derived^19–21^. The degrees to which the cell lines represent their parent tissues are still being investigated. In this paper, we have studied the present the electrical signatures of different cancer cell lines dielectric properties of breast cell lines and determined the difference in electrical properties of normal and cancerous breast cells.

## Materials and Methods

### Cell culture and reagents

Human breast cancer cells MDA-MB-231(HTB-26), Hs578T (HTB126), normal mammary epithelial cell MCF-10A (CRL-10317) and colon cancer cell line HT-29 were obtained from ATCC. Human breast cancer cells MCF-7 (300273) and T47D (300353) were obtained from Cell line service (CLS)-GmbH. MDA-MB-231, MCF-7 and HT-29 cells were maintained in DMEM (Hyclone, Cramlington, UK) complemented with 10% fetal bovine serum (FBS). T47D cells were maintained in RPMI (Hyclone, Cramlington, UK) complemented with 10% fetal bovine serum (FBS). MCF-10A were maintained in DMEM-F12 (In vitrogen) complemented with 15% FBS and 20 ng/mL human EGF recombinant protein (Sigma-Aldrich). All media were supplemented (Hyclone, Cramlington, UK), 100 U/ml penicillin/streptomycin (Hyclone, Cramlington, UK).

### Samples Preparation

For sample preparation, cells were seeded in triplicate in 12-well plates at a density of 4 × 10^5^ cells/well and placed in 37 °C incubator with 5% CO_2_ for 24 hours to allow cells to form homogeneous confluent monolayer. Prior to measurement, cells were washed three times in phosphate-buffered saline solution (PBS) at room temperature and then resuspended in 1 ml cell culture media.

### Instrument and Procedures

**Figure 1:**
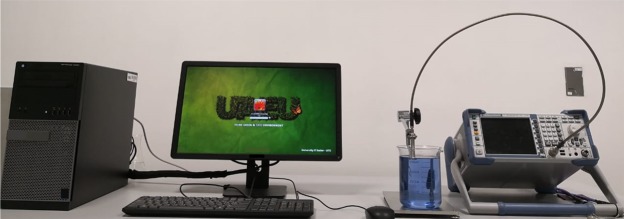
Microwave dielectric measurement setup consisting of an open ended coaxial probe, a vector network analyzer (VNA) and an external computer. Microwave signals generated by the VNA were transmitted via the coaxial cable to the probe tip submerged in the sample under test. Signals backscattered from the samples were analyzed using the DAK software installed in the computer to extract the dielectric parameters.

The open-ended coaxial probe (DAK-3.5 Dielectric Probe) developed by Speag was employed to determine the dielectric properties of cell line samples. An R&S®ZVL vector network analyzer (9 KHz to 13.6 GHz) and a Dielectric Assessment Kit software (DAK-3.5) were the key components of the measuring set up (Fig. 1). The DAK comprised of a DAK 3.5 dielectric probe with frequency range 200 MHz to 20 GHz, a probe stand and a highly flexible cable which connects the probe to the Vector Network Analyzer (VNA) port. An external computer, having DAK software installed, was connected to the VNA via Ethernet interface cable and the entire system was controlled by a Keysight connection suite. The overall functional frequency range of the system was from 200 MHz to 13.6 GHz.

**Figure 2:**
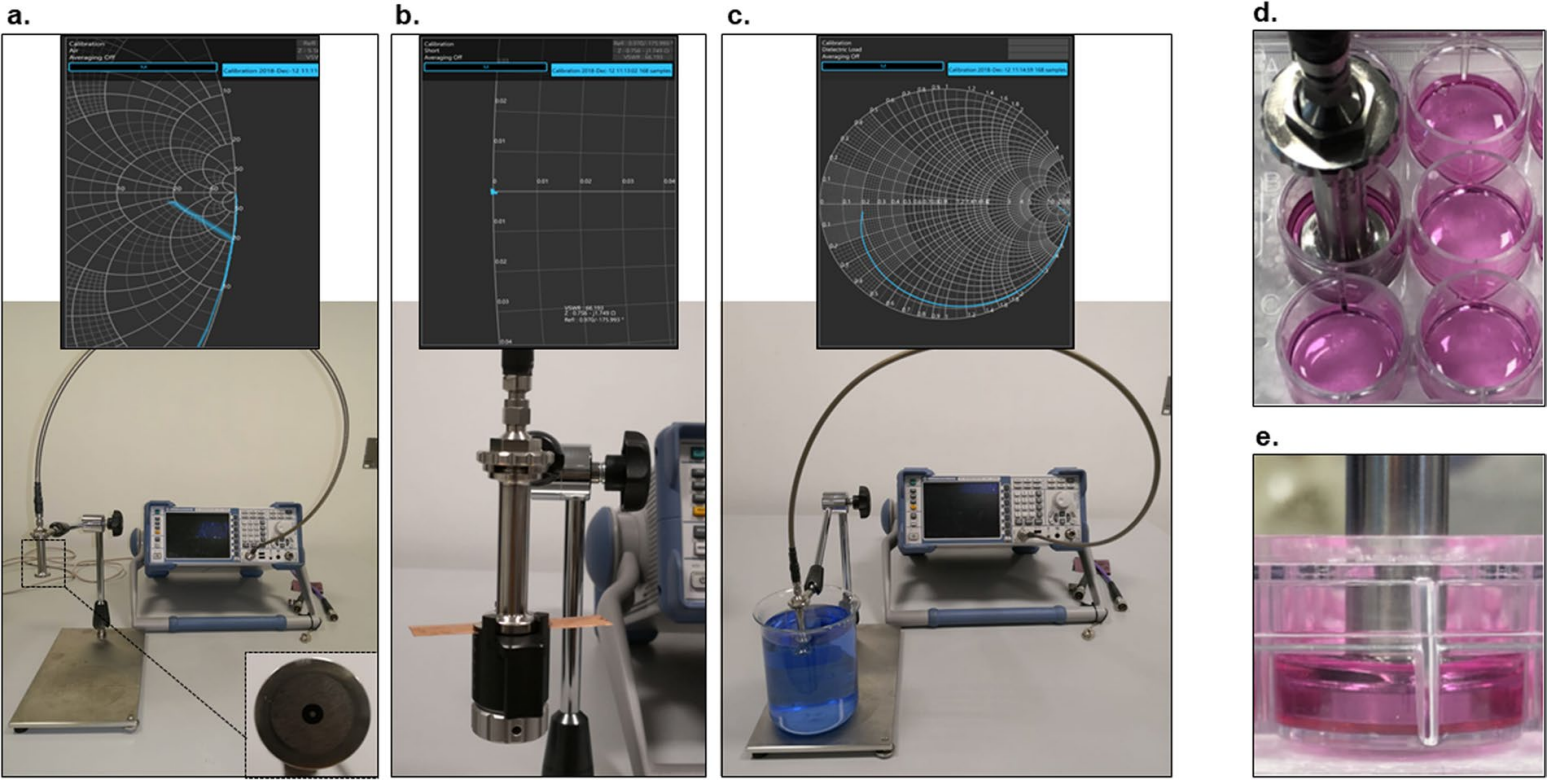
Calibration of measurement setup. Calibration was carried using open, short and load calibration. (**a**) Represents the open load calibration settings. Result depicted in Smith chart is shown in the upper panel. (**b**) Represents the short load calibration settings. Result depicted in Smith chart is shown in the upper panel. (**c**) Represents the load (deionized water) calibration settings. Result depicted in Smith chart is shown in the upper panel.

Prior to measurements, the DAK was kept in calibration mode and whole set up was calibrated using three independent standards: open, short and load. Calibration mode allows the user to enter the temperature of the load. The three calibrations were performed with the probe in air, short circuit mode and deionized water as depicted in Fig. 2a–c, respectively.

After calibration, the DAK was placed in measurement mode and the sample (cultured cell lines) to be measured was kept under the probe in such a way that the probe tip was slightly immersed in it without any air bubbles on the probe surface, and the probe kept at very close proximity from the base of the cell line plate, at about 0.75 mm. The prepared sets of samples were analyzed with and without culture media. For analysis without media, the solution was carefully removed by means of a sterile pipette. To avoid any contamination, the probe was wiped with ethanol in between measurements. It was also ensured that the probe or the cable was not moved during the measurements so as to avoid any kind of distortions in the amplitude and phase of the reflected waves through the cable. All measurements were acquired at room temperature in the 200 MHz-13.6 GHz frequency band. VNA computes the reflection coefficients at the probe tip and the DAK software converts it into dielectric parameters.

### Calculation of the dielectric properties of normal and breast cancer cell lines

Microwave dielectric characterization by open ended coaxial cable method serves as a low cost, easy and fast technique to identify the dielectric properties of biological samples. The way biological samples interact with microwave radiation is governed by its dielectric properties, which in turn are the result of cellular or molecular level chemical constituents of the samples. The interaction of microwaves with biological matter is attributed to two different processes: energy storage caused by dipole polarization in response to external applied field and energy dissipation when the dipoles do not align instantly with varying field. The dielectric properties are therefore represented in terms of complex relative permittivity as

1 $$\varepsilon =\varepsilon ^{\prime} -j\varepsilon ^{\prime\prime} $$

where, *ε′* is its real part representing the energy stored and ε*″* is its imaginary part representing losses.

2 $$\varepsilon ^{\prime\prime} =\sigma /\omega \,{\varepsilon }_{o}$$

where, $\sigma \,\,$is the electrical conductivity of the sample, *ω* is the angular frequency and *ε*_*o*_ is the free space permittivity.

Water, a polar molecule being the major chemical constituent of biological tissues contributes mainly to the interaction with electromagnetic radiation. The dielectric behavior of water molecules can be reasonably approximated using the well-known Debye relation^22,23^:

3 $${\varepsilon }^{\text{'}}(\omega )={{\varepsilon }^{\text{'}}}_{\infty }+\frac{{{\varepsilon }^{\text{'}}}_{s}-{{\varepsilon }^{\text{'}}}_{\infty }}{1+{\omega }^{2}{\tau }^{2}}$$

4 $${\varepsilon }^{\text{'}\text{'}}(\omega )=\frac{({{\varepsilon }^{\text{'}}}_{s}-{{\varepsilon }^{\text{'}}}_{\infty })\omega \tau }{1+{\omega }^{2}{\tau }^{2}}$$

where $\,{\varepsilon }_{s}^{^{\prime} }$, is the real static permittivity, and ${\varepsilon }_{\infty }^{^{\prime} }$ is the real high frequency permittivity and $\tau =\frac{1}{2\pi \,{f}_{r}}$ is the relaxation time; *f*_*r*_ is the relaxation frequency at which the dipole molecules fail to align with the field resulting in larger variations in real and imaginary parts of permittivity. At this particular frequency, real part tends to decrease and imaginary part shows a maximum value.

In addition to water molecules, cells contain other species such as free ions, carbohydrates, proteins and other organic chemical compounds. Ionic species contribute to energy dissipation and hence their behavior can be explained by adding an additional term in Equation (4) as

5 $${\varepsilon }^{\text{'}\text{'}}(\omega )=\frac{({{\varepsilon }^{\text{'}}}_{s}-{{\varepsilon }^{\text{'}}}_{\infty })\omega \tau }{1+{\omega }^{2}{\tau }^{2}}+\sigma /\omega {\varepsilon }_{0}$$

Similarly, the other species are also polar in nature and behave differently in an electric field. The effect of these species can be modeled by adding to these equations, appropriate parameters specific to their behavior.

Biological cell characterization using microwaves is indeed more reliable as the waves with these frequencies completely penetrate the cell membranes, leading to accurate identification of the dielectric nature of intra cellular contents.

### Statistical analysis

Statistical analysis were done using SPSS version 21 software. All statistical analysis were performed using one-way ANOVA test followed by Tukey’s HSD test. Significance for all statistical comparisons was set at p < 0.05 (*p < 0.05, **p < 0.005 and ***p < 0.001).

## Results and Discussion

**Figure 3:**
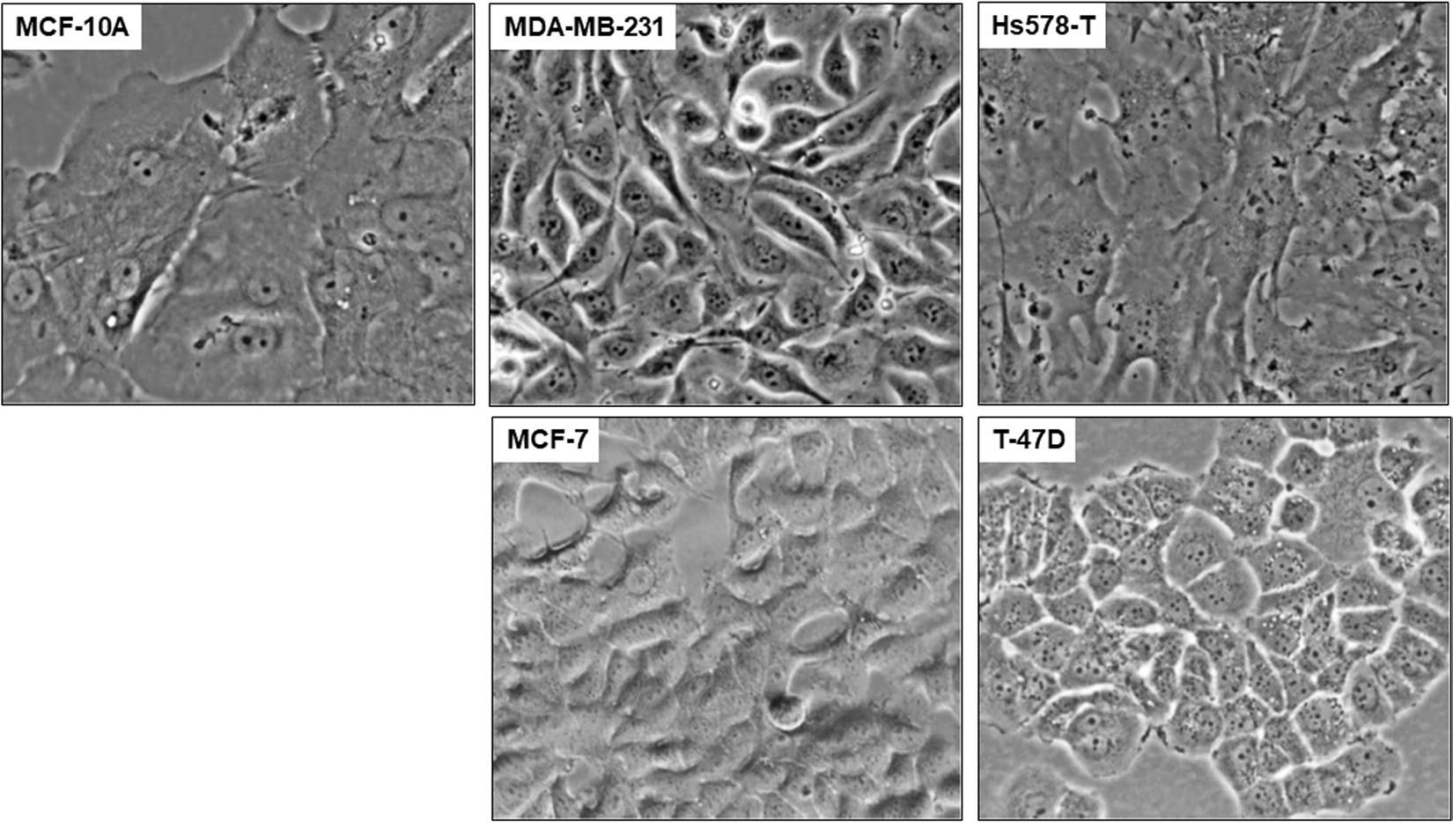
Morphology of normal and breast cancer cells. Prior to measurement, monolayer grown normal mammary (MCF-10A) and breast cancer cells (MDA-MB-231, Hs578T, MCF-7 and T47-D) cells were observed under EVOS XL Core Cell Imaging System (Life Technologies) at 400x.

In the present study, four types of breast cancer cell lines, two highly invasive and metastatic triple negative (MDA-MB-231 and Hs578T) and two less invasive and metastatic (T47D and MCF-7) breast cancer cell lines along with the non-tumorigenic breast cell line (MCF-10A) have been characterized *in vitro* for their dielectric properties. Figure 3 shows the morphology of these samples prior to measurement.

**Figure 4:**
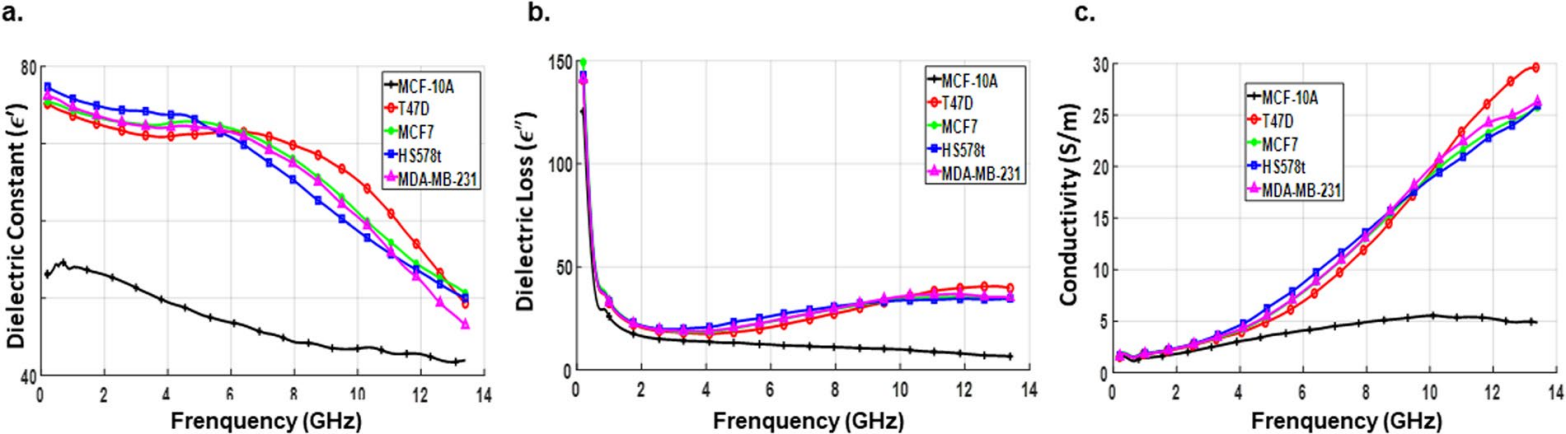
Measured dielectric data of breast cell lines with the culture medium. Average values of dielectric constant (**a**), dielectric loss (**b**) and conductivity (**c**) as a function of frequency for each of the prepared samples (normal and cancerous breast cell line) measured with the culture medium. Data are representative of three independent experiments carried out in triplicate.

**Figure 5:**
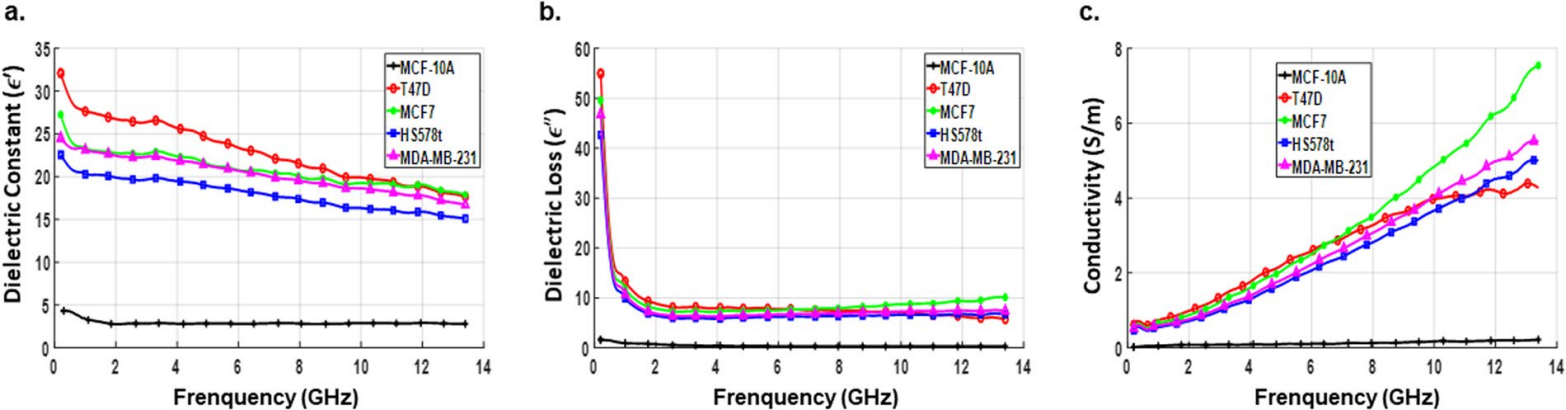
Measured dielectric data of breast cell lines without the culture media. Average values of dielectric constant (**a**), dielectric loss (**b**) and conductivity (**c**) as a function of frequency for each of the prepared samples (normal and cancerous breast cell line) measured without the culture medium. Data are representative of three independent experiments carried out in triplicate.

With three replicates per sample, a total of 39 readings per experiment were taken from the samples including the culture medium. Experiment was repeated three times at different days and with freshly thawed cells each time. The dielectric data corresponding to each sample were analyzed and averaged. The permittivity and conductivity values of the five cell lines evaluated with and without the culture media are shown in Figs 4 and 5, respectively. Dielectric spectroscopic studies revealed three basic mechanisms namely $\alpha ,\,\beta$ and $\gamma$-dispersions that characterize the frequency dependence of dielectric properties of biologicals systems^16^. The α-dispersion falls in the lower frequency range (kHz range) and is related to ionic diffusions at cell membranes. The β-dispersion which arises from cell membrane polarization occurs at the medium frequency range (lower MHz range). Finally, the $\gamma$-dispersion belongs to the higher frequency range (GHz range) at which the waves propagate across the cell membrane causing polarization of polar molecules.

In this study, β-dispersion and most of the $\gamma$-dispersion range are included in the desired operating frequency band. We observed that in the presence of culture medium, all cancer cell lines showed a higher dielectric constant, compared to non-tumorigenic cells, especially in the lower part of the measurement spectrum among which Hs578T showed the highest value. In the β-dispersion region of the frequency spectrum, dielectric measurements exhibited higher polarization effect for all types of cancer cell lines. This might be due to significant membrane resistance which inhibits wave propagation into the cell.

Between 4–7 GHz, the dielectric response was dominated by broad asymmetric peaks (points of inflection) in the spectra of dielectric constants of cancer cell lines. These inflection points denoted critical transition points after which the dielectric response reversed its trend, with T47D showing the highest value followed by MCF-7, MDA-MB-231 and Hs578T. In the $\gamma$-dispersion range, cell membrane resistance dropped allowing the waves to penetrate the cell. As a result, the dielectric constant of all cell lines showed a decreasing trend with frequency and at frequencies above 7 GHz, relaxation type behavior started to appear and the dielectric constant decreased sharply with increase in frequency and dropped to approximately *ε′* = 50 at 13.6 GHz. This type of behavior clearly indicates the high cytoplasm conductivity of the cell upon wave penetration. Hence, each cancer cell line can be differentiated based on their respective inflection point and dielectric responses. The point of inflection representing the frequency at which change in the behavior of each cell line is noted was 6.0, 4.7, 4.7 and 4.3 GHz for T47D, MCF-7, MDA-MB-231 and Hs578t cells, respectively. Dielectric constant of the non-tumorigenic cell line MCF-10 A, on the other hand, had much lower value, did not show inflection point and varied over the measurement spectrum at a relatively lower rate than that of cancer cell lines. Also, dielectric loss as well as conductivity exhibited a similar trend with respect to cancer cell line type but showed larger values to increase in frequency except for the non-tumorigenic cell line the values of which decreased at higher frequencies. As it is shown in Table 1, statistically significant differences were observed between the dielectric properties of non-tumorigenic (MCF-10A) and cancerous breast cell lines (T47D, MCF-7, MDA-MB-231 and Hs578T). These differences may be due, although not solely, to the high water content present in cancer cells. Indeed, it has been shown that cancer cells have higher content than normal cells^24,25^. The frequency variation of dielectric parameters follows the Cole–Cole pattern^22,23^. The interactions of biological cells with microwaves are translated to relaxations and resonances of dielectric parameters. Our results are consistent with other published results for bulk tissues^13^.

**Table 1:** Average and standard deviation values of dielectric constant and conductivity of breast cancer cell lines at specific frequencies.

Frequency	T-47D		MCF-7		HS578t		MDA-MB-231	
	ε′	σ (S/m)	ε′	σ (S/m)	ε′	σ (S/m)	ε′	σ (S/m)
900 MHz	73.87^**^ ± 0.63	1.79^**^ ± 0.01	74.45^**^ ± 0.39	1.88^*^ ± 0.03	75.99^**^ ± 0.53	1.83^***^ ± 0.004	74.85^***^ ± 0.49	1.80^***^ ± 0.04
2.5 GHz	71.74^**^ ± 0.71	2.64^*^ ± 0.03	72.81^**^ ± 0.57	2.72^*^ ± 0.03	74.36^**^ ± 0.94	2.79^**^ ± 0.01	72.80^***^ ± 0.58	2.72^***^ ± 0.02
6.5 GHz	71.45^**^ ± 0.53	8.08^*^ ± 0.65	71.26^**^ ± 0.69	9.07^*^ ± 0.53	69.58^*^ ± 1.59	9.98^**^ ± 0.21	70.75^***^ ± 0.20	9.13^*^ ± 0.52
10 GHz	65.24^*^ ± 2.20	19.34^*^ ± 0.84	61.09^*^ ± 2.19	19.10^*^ ± 0.75	58.73^**^ ± 0.56	18.78^*^ ± 1.71	60.47^*^ ± 1.83	19.81^***^ ± 0.15
13 GHz	51.23^*^ ± 2.05	29.36^*^ ± 1.50	51.74^*^ ± 1.42	25.02^*^ ± 1.89	49.67^*^ ± 3.24	24.84^***^ ± 0.26	47.97^**^ ± 0.43	25.57^**^ ± 1.19

On the other hand, in the absence of culture medium, cancer cell lines displayed lower dielectric values varying at a slower rate over the measurement frequency spectrum. The non-tumorigenic cell line exhibited the least dielectric response with slight or no variation with respect to frequency. Based on the results obtained from cell lines without the media, the contrast in the dielectric constant and conductivity of cancerous and normal cell lines was determined. The dielectric and conductivity ratio, at three specific frequencies, between normal and cancerous cells are summarized in Table 2. A considerable contrast was present throughout the measurement frequency range with the dielectric constant following a decreasing trend with frequency while the conductivity showing an increasing trend with frequency. It is noteworthy to mention that slight variations in the measurements between different cell lines may result from the type of culture media used. In order to avoid such variations, we recommend, that after PBS wash, to re-suspend the cells in the same media. However, measurement carried out without media eliminates such variations.

**Table 2:** Ratio of dielectric constant and conductivity between normal (MCF-10A) and cancerous breast cell lines.

Frequency (GHz)	MCF-10A vs. T-47D		MCF-10A vs. MCF-7		MCF-10A vs. MDA-MB-231		MCF-10A vs. Hs578-T	
	ε′	σ (S/m)	ε′	σ (S/m)	ε′	σ (S/m)	ε′	σ (S/m)
4	~9.1	~19.8	~7.9	~18.0	~7.8	~15.6	~6.9	~14.6
8	~7.6	~25.5	~7.1	~27.4	~6.9	~23.9	~6.1	~22.0
12	~6.5	~21.5	~6.6	~32.1	~6.1	~25.6	~5.5	~23.2

**Figure 6:**
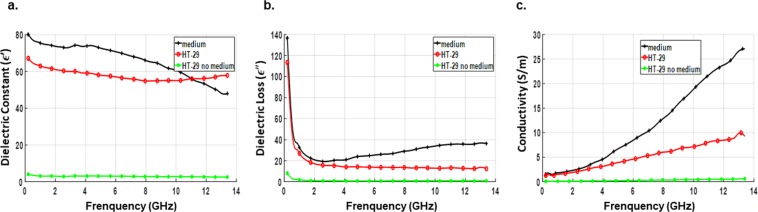
Comparison of dielectric properties of colon cancer cell lines and the culture medium. Average values of dielectric constant (**a**), dielectric loss (**b**) and conductivity (**c**) as a function of frequency for each of the prepared samples; samples of HT-29 colon cancer cell line were analyzed with and without the culture medium. Data are representative of three independent experiments carried out in triplicate.

**Figure 7:**
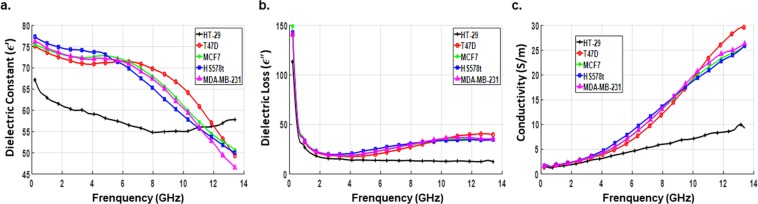
Comparison of dielectric properties of colon and breast cancer cell lines. Average values of dielectric constant (**a**), dielectric loss (**b**) and conductivity (**c**) as a function of frequency for each of the prepared samples of colon and breast cancer cell lines.

Next, we analyzed the dielectric properties of the human colon cancer cell line HT-29. Figure 6 depicts the comparison of dielectric properties of colon cells with and without culture medium. Compared to the medium, colon cell lines showed a different behavior in response to microwave signals with relatively small variation at the higher frequency band. The average values of dielectric constant and conductivity for the colon cells at 5 specific frequencies are listed in Table 3. Next, we further run comparative analysis of the dielectric behavior of colon and breast cancer cell lines. As shown in Fig. 7, in contrast to breast cancer cells, the dielectric response of colon cancer cells did not show any inflection points within the frequencies tested. At lower frequencies, the dielectric polarization effect observed for colon cancer cell lines was lesser compared to that of breast cancer cell lines. Further, the rate of variation of dielectric constant as well as dielectric loss and conductivity with increase of frequency was even less which revealed the higher membrane resistance and a lower cytoplasm conductivity of colon cancer cell lines in comparison to breast cancer cell lines. This suggests that each type of cancer cell line might have a unique microwave signature and can be identified based on it.

**Table 3:** Average and standard deviation values of dielectric constant and conductivity of colon cancer cell lines at specific frequencies.

Frequency	Colon cancer cell line (with medium)		Colon cancer cell line (without medium)	
	ε′	σ (S/m)	ε′	σ (S/m)
900 MHz	63.37 ± 4.26	1.48 ± 0.09	3.15 ± 0.32	0.10 ± 0.005
2.5 GHz	60.39 ± 3.52	2.19 ± 0.11	2.85 ± 0.29	0.12 ± 0.007
6.5 GHz	56.39 ± 4.16	4.96 ± 0.35	2.96 ± 0.25	0.26 ± 0.01
10 GHz	55.03 ± 5.16	7.14 ± 1.03	2.77 ± 0.19	0.42 ± 0.02
13 GHz	57.71 ± 6.88	9.87 ± 3.11	2.53 ± 0.17	0.56 ± 0.02

## Conclusion

In this study, we determined the dielectric response of breast and colon cell lines. The comparison of dielectric properties of breast cell lines revealed a significant dielectric contrast between normal and cancer cells. The dielectric response exhibited relaxations and resonances, which can be attributed to the dielectric relaxations of cells. Our analysis shows that microwave characterization of cell lines is reliable and has a strong potential in biomedical applications like cancer detection and designing electromagnetic models of healthy and tumorous tissues.
